# 828. Short- and Long-Term Metabolic Changes in Virologically Suppressed Patients Switching from TDF to TAF Containing Antiretroviral Therapy

**DOI:** 10.1093/ofid/ofab466.1024

**Published:** 2021-12-04

**Authors:** Jason J Schafer, Matty Zimmerman, Ciara E Walshe, Jessie Cerankowski, Ayako Shimada, Scott Keith

**Affiliations:** 1 Jefferson College of Pharmacy, Philadelphia, PA; 2 Thomas Jefferson University, Hellertown, PA

## Abstract

**Background:**

Switching from tenofovir disoproxil fumarate (TDF) to tenofovir alafenamide (TAF) containing antiretroviral therapy (ART) may negatively influence weight, cholesterol, and atherosclerotic cardiovascular disease (ASCVD) risk. The timing, duration, and extent of these changes and their definitive associations with TAF remain unclear.

**Methods:**

This retrospective observational study evaluated weight, body mass index (BMI), cholesterol, and ASCVD risk score changes in virologically suppressed patients living with HIV infection (PLWH) who switched from TDF to TAF without switching any other ART regimen components. Adult patients on TDF and no HIV viral load values > 200 copies/mL for ≥ 2 years prior to and following a TAF switch were included. Body weight, BMI, cholesterol and other variables were collected for the 2 years before and after the switch. The Wilcoxon signed-rank test compared median values for each measurement pre and post switch in a univariate analysis. Longitudinal linear mixed effects models evaluated changes for each outcome measure at 1 and 2 years after the switch. Models were built with random effects for patients and included covariates such as time on TAF, age, sex, race, time with HIV, diabetes, smoking status, and concomitant medications associated with weight gain or loss.

**Results:**

A total of 86 patients met study criteria (table 1). In the univariate analysis, there were significant increases in weight, BMI, total cholesterol, LDL, HDL, triglycerides, and ASCVD risk scores 2 years after switching to TAF (each p ≤ 0.05, table 2). However, after controlling for covariates, only the increases in total and LDL cholesterol were associated with switching to TAF and significantly different from expected changes predicted in the linear model. In terms of weight gain with TAF, patients gained an average of 4.3 pounds in year 1 and 3.8 pounds in year 2 after the switch. Neither of these increases were statistically different from the expected changes in weight predicted in the linear model (3.1 pounds/year, 95% CI: 1.6-4.6).

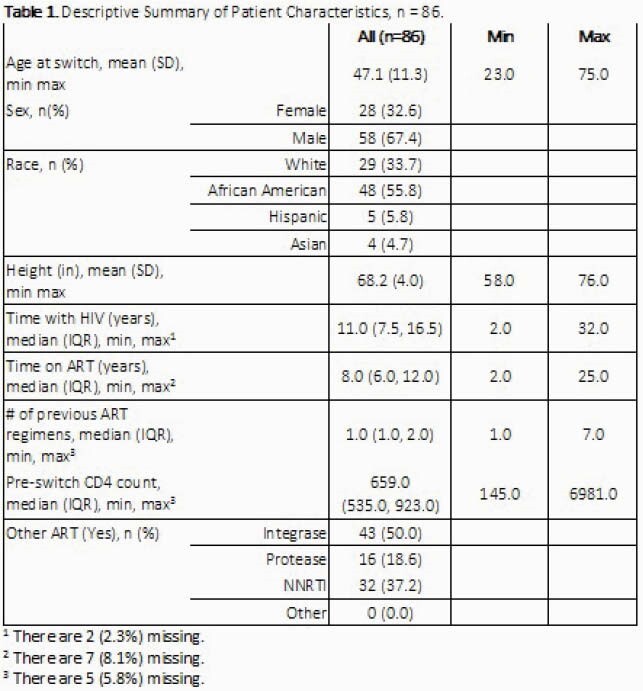

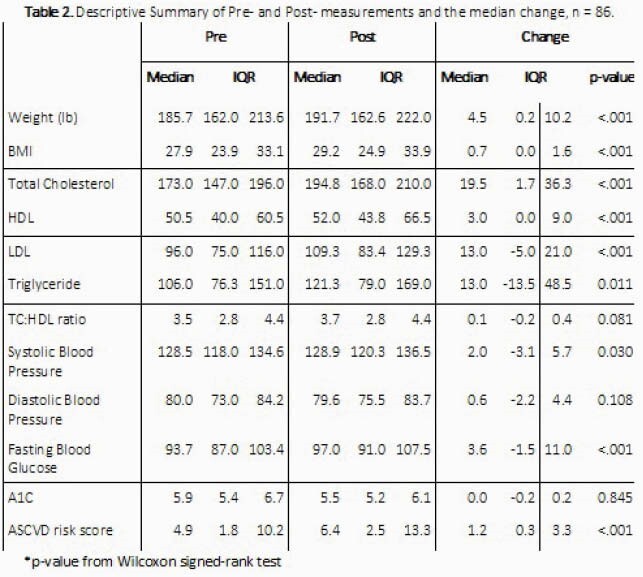

**Conclusion:**

Despite observing significant increases in weight, BMI, cholesterol and ASCVD risk scores after switching to TAF, only the changes in cholesterol were significantly associated with TAF and different from changes expected in PLWH over time.

**Disclosures:**

**Jason J. Schafer, PharmD, MPH**, **Gilead** (Research Grant or Support)**Merck** (Advisor or Review Panel member, Research Grant or Support)**ViiV** (Advisor or Review Panel member)

